# *“It’s a balance of just getting things right”:* mothers’ views about pre-school childhood obesity and obesity prevention in Scotland

**DOI:** 10.1186/1471-2458-14-1009

**Published:** 2014-09-27

**Authors:** Flora Douglas, Julia Clark, Leone Craig, Jonina Campbell, Geraldine McNeill

**Affiliations:** Public Health Nutrition Research Group, Rowett Institute of Health and Nutrition, University of Aberdeen, Polwarth Building, Foresterhill, Aberdeen, AB25 2ZD Scotland, UK; University of Aberdeen, NHS Grampian, Aberdeen, Scotland

**Keywords:** Young children, Obesity, Mother’s views, Weight management, Qualitative research, Scotland

## Abstract

**Background:**

The high prevalence of childhood obesity is a concern for policy makers and health professionals, leading to a focus on early prevention. The beliefs and perspectives of parents about *early* childhood obesity, and their views and opinions about the need for weight management interventions for this age group are poorly understood.

**Methods:**

A formative qualitative focus group study with parents of pre-school children took place in eight community-based locations throughout North-East Scotland to explore their ideas about the causes of early childhood obesity, personal experiences of effective weight management strategies, and views about the format and content of a possible child-orientated weight management programme. Study participants were recruited via pre-school nurseries.

**Results:**

Thirty-four mothers (median age 37 years) took part in the study, but only two believed their child had a weight problem. Participants (who focussed primarily on dietary issues) expressed a strong sense of personal responsibility to *‘get the balance right’* regarding their child’s weight, and were generally resistant to the idea of attending a weight management programme aimed at very young children. At the same time, they described a range of challenges to their weight management intentions. These included dealing with intrinsic uncertainties such as knowing when to stop ‘demand feeding’ for weight gain, and judging appropriate portion sizes - for themselves and their children. In addition they faced a range of extrinsic challenges associated with complex family life, i.e. catering to differing family members dietary needs, food preferences, practices and values, and keeping their ‘family food rules’ (associated with weight management) when tired or pressed for time.

**Conclusions:**

The findings have important implications for health professionals and policy makers wishing to engage with parents on this issue, or who are currently developing ‘family-centred’ early childhood weight management interventions. The challenge lies in the fact that mothers believe themselves to be the primary (and capable) agents of obesity prevention in the early years – but, who are at the same time, attempting to deal with many mixed and conflicting messages and pressures emanating from their social and cultural environments that may be undermining their weight management intentions.

## Background

Parents are commonly regarded as those best placed to support obesity prevention and reduction efforts for young children [1, 2]. At the same time, they are also viewed as the main culprits (mothers in particular) if a child becomes overweight or obese [3, 4]. Covney argues that ‘parenting’ in general has become the focus of a variety of discourses in contemporary society, in which nutrition commonly features, with parents’ proficiency (as parents) commonly judged by themselves and others against benchmarks determined by expert opinion [5]. This subject and perspective has attracted significant media attention over recent years, with an increasing number of published newspaper articles focused on the (de)merits of parents in relation to the weight status of their children [6, 7].

There is high level of policy concern about childhood obesity rates in the UK. The latest figures released by ISD in Scotland suggest that although the BMI distribution of children at school entry (around five years of age) has remained broadly similar over the period 2001/02 to 2012/13, around 21% to 23% of children (one in five) are at risk of overweight and obesity [8]. The Scottish Government views obesity as “*a real risk to the health of the population in Scotland and its ability to meet its overarching purpose of sustainable economic growth because of the burden of disease that accompanies*” it, and has, amongst a range of obesity related objectives, a particular focus on children and families as targets for intervention [9].

However, there is a dearth of knowledge about parents of very young children’s perspectives associated with programmes or interventions targeting this age group in the UK. It is well established that, *before* embarking on the design of any new health promotion programme or intervention or programme, it is important to understand the worldviews and values of those individuals or groups intended to benefit from them [10–12]. Indeed, individuals willingness to support (or engage with) a particular course of action is determined by the relationship that exists between their beliefs about the causes of a particular health or social problem, and, what they think could or should be done to address it [13, 14]. It has been argued that traditional public health nutrition approaches that have been based largely on realistic/reductionist epistemology have failed to address our nutrition-related population health problems, and that researchers, policy makers and practitioners need to consider such problems through structural and agential perspectives in order to develop and deliver more effective interventions [15].

In this study parents’ and health professionals’ views and perspectives about pre-school childhood overweight and obesity were sought by a local health board in order to develop a weight management programme orientated towards parents of children under the age of five living in the North East of Scotland. This paper focuses on mothers perspectives’ about the nature and causes of childhood obesity, their views and experiences of managing their child’s weight, and, about effective weight management strategies for this age group. This included exploring the perceived role of health professionals associated with their endeavours to raise a “healthy weight” child during infancy.

## Methods

Parents and carers of children aged 3–4 years old were sent a letter with information about the study via thirty-six pre-school nurseries in North-East Scotland. The nurseries were selected to ensure that parents living in areas of high and low socio-economic deprivation, and both urban and rural locations could be invited. Thirty-four mothers agreed to participate in the research, and took part in a total of nine focus group discussions, which took place over a period of two months in a variety of community-based locations across the region. Written informed consent was gained from all participants.

The study was conducted using principles and techniques found in Grounded Theory approaches [16]. A topic guide was developed and used to guide the discussions and enabled the researchers to combine inductive and deductive reasoning to generate and analyse the data. Topics explored during the focus groups included: a. parents’ views about the prevalence of obesity and overweight in Scottish children in general, b. their views about their own child’s weight status (including whether they had ever been told by a health professional that their child was overweight, c. factors they believed were responsible for childhood overweight and obesity, d. their views about parents’ role in child weight management and e. their views about the relevance/utility of weight management programmes for this age group. The focus groups lasted between one and two hours. Every attempt was made to ensure that all parents participated in the discussions and to look for alternative perspectives during the discussions. In all cases two researchers were present during the focus groups; one person who acted as facilitator and the other the scribe. All interviews were audio-recorded and transcribed verbatim.

The data was analysed thematically [17] and NVivo version 10 (QSR) (computer assisted data analysis software) was used to support data management and retrieval. Initially a sample of interview transcripts were read and re-read independently by two researchers to identify the key concepts and themes and a draft coding index was drawn up. The researchers met to discuss their initial analysis: areas of difference were identified and areas of disagreement were resolved. The final version of thematic index was also agreed through discussion and all transcripts were coded in the NVivo database. Memos and notes of emerging themes, issues and patterns were also recorded within NVivo and were referred to during the analysis. Constant comparison method was used throughout to ensure consistency of coding and assigning of coded data to the emergent themes and categories, and, to ensure that possible new themes were not being overlooked. Every attempt was made to search for disconfirming data within the data set. Furthermore, individual case data (within each focus group) was also examined to determine the consistency of viewpoints within those individual accounts, or possible deviations or contradictions from an individual’s original viewpoint that may have occurred during the discussions. The data were also considered for the possibility of dominant and/or marginalised viewpoints. Anonymised, illustrative quotes are used in the results section to illustrate the key themes.

Ethical approval was sought and granted for this research from the North of Scotland Research Ethics Committee. (Reference number 2012CH005). The manuscript was written in accordance with the RATS qualitative research review guidelines.

## Results

Thirty-four mothers with children under five years with a median age of 37 years took part in the study (age range 23–42 years). They came from a range of socio-economic backgrounds (i.e. ranging from manual occupations, professional, self employed and full time caring backgrounds), and over half had more than one child. All but two parents indicated that they *did not believe their child had a weight problem* (See Table 1 for participant details).

**Table 1:** **Characteristics of the parents participating in focus groups and their pre-school child (n = 34)**

Age (yrs)	Age when completed education (yrs)	Occupation	Total children	Sex of child	Weight concerns (own child)
40	≥19	Manager with Local authority	2	M	No
33	≥19	Business support	3	F	No
40	17	Management Accountant	2	M	No
33	≥19	Children's nurse	2	F	No
37	≥19	Alumni development Exc	2	F	No
37	≥19	Teacher	2	F	No
41	≥19	Part time dental hygienist	2	M	No
38	18	Part time production planner	2	F	Yes
40	≥19	Nurse	2	F	No
N/G	N/G	N/G	N/G	N/G	N/G
32	≥19	Police officer	2	M	No
36	HNC at 20	Clerical, FTM	1	F	yes
39	≥19	Nurse	2	M	No
37	≥19	PA	3	F	No
37	≥19	Physiotherapist	2	M	No
40	≥19	Development marketing manager	2	F	No
37	17	Care attendant	1	F	No
37	≥19	Teacher	3	F	No
33	≥19	Staff nurse	3	M & F	No
38	≤16	Activities co-ordinator, FTM	2	M	No
41	≥19	Primary school teacher	2	M	No
37	≥19	Translator	2	F	No
37	≥19	FTM	3	F	No
N/G	N/G	N/G	?	F	No
42	≥19	Payroll, FTM	3	M	No
40	≥19	Offshore tech, FTM	4	F	No
38	≤16	Part time factory worker	2	M	No
28	≥19	Sales assistant	1	M	No
N/G	18	Clerical/admin	2	M	No
39	≥19	Staff nurse	2	M	No
40	≥19	Optometrist	1	F	No
39	≥19	Primary teacher	2	F	No
35	≥19	Part time cleaner	2	F	No
23	18	Property developer	1	F	No

N/G = Not Given.

FTM = Full time mother.

The key emergent themes included: (a) *problematic parenting* and *individual (ir)responsibility* as the perceived primary cause of obesity, ‘(b) *responsible, balanced parenting’,* as the key means by which overweight and obesity was prevented in this age group, and (c) the existence of *multiple intrinsic uncertainties and extrinsic familial and social challenges undermining mothers’ food- related weight management intentions*.

### A. Perceptions of causes and means of preventing childhood obesity

#### From problematic others to deficient self

The most prominent theme to emerge about the causes of childhood obesity (which overshadowed all other explanations) was that of *parental failure.* Many examples of perceived parental wrongdoing were presented during the discussions. Those focused primarily on parents’ failure to provide appropriate food at family mealtimes, and, to manage their children’s food intake. *Other* parents were initially considered as those not knowledgeable about healthy eating; not having skills or knowledge about how to ‘cook from scratch’ (compared to times past); not knowing how to shop and budget; not being motivated enough to cook, or being prepared to give sufficient effort to planning and cooking meals. This example was typical:

*“As well education because there will be a percentage of the population that it’s culturally, they grew up on fried food and that they don’t know that that’s not healthy, it’s not their fault it’s just they don’t know.”* (P7)

Providing insufficient opportunities for their children to be physically active or, lacking ability to limit or restrict their children’s sedentary activities such as computer use and TV viewing were also discussed, but far less often than food issues.

It is important to note that these thoughts (about other parent’s ‘deficiencies’) were volunteered towards the start of each discussion. However, a number of parents who initially inferred they were good, capable parents at the start of their discussion group, started to admit (at some later point) they were not always able to pursue their own healthy eating ideals and talked about *themselves as deficient* in some sense. As one parent started to admit to their struggles to be a ‘good parent’, others started to disclose their own perceived shortcomings. Admissions of taking food shortcuts, allowing bad foods and treats, and using convenience foods when they were tired after work, or lacking time then became commonplace,

*“It’s not a deliberate thing but convenience food is easy and I rely on it as well, in fact, I mean, my children are kinda healthy weight range, but I still have habits that I don’t like that I’m giving them, like I’m busy and I’m tired and I come home from work and I know the foods that they’ll eat is like chicken dippers and sometimes do fried potatoes and things like this .. and I’m thinking as well it’s good for them cause it’s got high calories and I want to get some meat on their bones, but it’s probably not good habits for them for the rest of their life, but it’s the way that we cope with not having a lot of time.”* (P01)

#### Structural factors

Participants talked about structural causal factors, but downplayed these in favour of the parental deficit explanations. These narratives centred largely on the changes they noticed in their food environments compared to when they were growing up, such as the cost of food, reduced time available (for mothers) to prepare meals, the widespread availability of unhealthy and convenience food, and perceptions of supermarkets encouraging people to eat too much. This was discussed in fatalistic terms and viewed as something that individuals just *‘had to be strong’* about dealing with, as illustrated here:

*P11: …, “we were very much marketing for children and the whole pester power in the supermarkets, and I’m probably guilty of ‘oh whatever, just something small’ to keep them going round the shops, but the children are influenced and then they want the Milky Bar and they want the..”.*

*P12 “So they are slightly hypocritical aren’t they in that sense, the supermarkets, cause they’re all for this, you know, ‘this week we’ve got all the five veggies and they’re all 50p each’ but actually when you get to the checkout…”*

*P11: “Yes there’s the Kinder Surprise.”*

*P13: …”there’s the Kinder Surprise and top to bottom isn’t it, and of course you're standing for ages at the checkout”*

Problematic aspects of their children’s levels of physical activity were mentioned here too, but to a much lesser extent. In some groups, this issue only was discussed after probing.

### B. Perceived solutions

#### Responsible, balanced parenting

When we asked parents what they thought was required to raise a healthy weight child *parental responsibility to get the balance right* emerged as the key theme. Within this theme, sub-themes of culinary capabilities and skills, parenting styles and approaches, personal agency and autonomy emerged as important.

All discussions contained lengthy accounts of cooking tips and techniques and detailed descriptions of favourite family meals. Weaning practices and family food routines were also described. References to foods and meals participants’ mothers had cooked for them as children, along with explanations of their efforts to mimic or replicate their mother’s culinary practices. The extent to which these descriptions featured in the discussions was striking, and partly responsible for the relatively long average length of the discussions.

Good parenting skills and exhibiting good personal food behaviours (as parents) was also considered an essential to this balancing. Two different ideas about being good [weight management] parents emerged, i.e. those who restricted their infant’s food choices day-to-day, and those who claimed they deliberately *did not* restrict them. The ‘non-restrictors’ talked about training their children to choose the ‘right sorts’ of foods by providing all types of foods, ‘good’ and ‘bad’, in moderation. A few justified this approach by suggesting it was their responsibility to prepare their child to make good decisions about their food and diet in the future. The notion of deliberately introducing foods (such as chocolates and sweets) to a child’s diet, hoping that they would learn self-control, commonly emerged, as illustrated here:

*“ I don’t feel depriving my child or just saying absolutely categorically no to everything, I think that would then, yeah you would like go berserk if… but I think there’s the whole ‘banners’, at one would she know anything about chocolate, no, she has no clue, she’s absolutely no clue. I mean, I started giving her dark chocolate at two and it would be… and she actually does have chocolate but it’s all in moderation, …So I think it’s a balance, it’s a balance of just getting things right.”* (P22)

This approach contrasted sharply with those other parents who placed more emphasis on restricting and controlling their child’s food intake day-to-day. Those parents talked about the importance of allowing their child to eat only ‘healthy foods,’ and often talked about their ‘food rules’. Those who described this type of approach also talked a lot about daily struggles they had with their children in their efforts to keep to the ‘rules’.

Related to this ‘balancing’ was the great value participants placed on their autonomy and personal judgement in relation to their child’s weight status*.* As already indicated, the vast majority of our participants did not believe their child had a weight problem; indeed, having a ‘chubby’ child was viewed as a positive thing by many; needing *‘a little bit of extra ‘*padding’ to cope with active play, illness and ‘growth spurts’. It was notable throughout the discussions, that participants discussed BMI scores or a score derived from a centile chart, with varying degrees of scepticism. A few mentioned using BMI information when making judgements about their child’s size, but described using many other factors to reach conclusions about it: such as how their child compared to other children their age, their clothing size, their mood and behaviour, amongst a range of other things. Participants also reported receiving (what they had experienced as) conflicting advice from health professionals about their child’s weight. Some participants indicated that they had learned to more or less ignore that advice, highlighted here:

*“Yeah, I didn’t really pay much heed to it (the growth reference score given to them by their health visitor) if I’m honest. I breastfed both my children and I think as a mother’s instinct you just know if they’re feeding well, if you’re happy, if you think that they’re gaining weight, you know”* (P07)

We also noted that despite their concerns that their child would put on weight in the future, almost all were extremely resistant to the idea of the need for a health visitor-led weight management programme aimed at helping families with overweight pre-school children. They asserted that they and other parents would not use it. A strong theme to emerge here was the desire not to relinquish control of something they believed was their own responsibility to a health professional, illustrated here:

P34: “*Well, it sounds good but then people won’t like it will they, parents won’t like it”*

P33: “*I think a lot of parents don’t like being told…”*

P34: “*Yeah..”*

P30: “…*generally, like, they’re seeing it as somebody taking the control, you know, they don’t want to be told what they have to feed their children”.*

P33: *“I don’t think they’d like it at all. I don’t think you would get people to join a programme, you know, if they were overweight, I think the parents would just say ‘no we’re not doing it’”*.

Many also talked about parents being unwilling to have their competence as parents questioned, or be judged as failures by having to engage in such a programme.

### C. Intrinsic uncertainties and external challenges

#### Problematic portion size

There was a strong theme of confusion and anxiety surrounding age-appropriate portion sizes for pre-school age children. One mother talked about learning from the TV that she should have been feeding her two-year-old child much smaller amounts (of pizza) than she had been doing. This confession resulted in other parents in the group expressing surprise, anxiety and agreement that they too had been unwittingly overfeeding their children, as illustrated by this exchange:

P09: *“It’s actually unbelievable, I’m trying to think, there was a thing on telly and it was telling you about kids, maybe a food thing, and they showed you what a child should be eating like and it was one slice of pizza and I thought ‘oh my God’ you know, my three year old would… you know, she would eat two or three bits.”*

P10: “*That’s all they should have at a meal is one slice of pizza?”*

P09: *.”..it was one slice of pizza was the correct calories and everything, you know..”.*

P10: “*Gosh”.*

P09: “*And I thought ‘oh my goodness’ so you know, probably I do give them maybe… I probably give them too much but then I’m not expecting them to eat it all, you know, so.”*

P10: “*But I’ve never seen anywhere that shows what a portion size should be for kids…”*

P09: “*No”.*

P10: “*…and if you buy, you know the likes of that… I mean, I’ve got the… is it the dippers, dip in cheddar, .. and I think well that’s a portion kinda thing and you can add on fruit and other stuff, but if you’re cooking, if you just bought, like, the plastic plates out of Asda or Tesco, that’s her plate, it’s like ‘so do we fill that plate or should it be less?”*

INT: “*Okay”.*

P10: “*And that’s quite hard cause if you’re kinda dishing up it’s… what’s a portion size for a kid and as they’re growing, she gets the same portions now she did when she was two, I think ‘oh actually…’ [laugh] so did I overfeed her at two or [laugh] am I underfeeding now?”*

P11: “*My two get the same size and that’s them three and six, you know…”*

P08: “*So do mine”.*

#### Mixed messages: environmental and social

Others talked about the mixed signals they picked up from their environment (i.e. from supermarkets and cafes) about food portion size. They talked about everything “being bigger nowadays” (compared to when they were growing up), e.g. cups, plates, serving sizes of crisps and drink cartons which made it hard to judge what a normal portion size was - for themselves and their children. Some mothers thought they were probably overeating themselves due the large serving sizes they perceived were commonly offered in cafes and restaurants compared to when they were children.

Some talked also about feeling pressure from other people (friends and family members) about how much to feed their child. This emanated from their observations of how well their child was thriving (or not). Health professionals were also mentioned as being responsible for some of this confusion, due to their perceived focus and concern about underweight in early infancy. A few talked about this causing them to introduce their babies to ‘bad’ foods or excessive amounts of food to help them to grow faster.

Others talked about finding it difficult to know or recognise when they should stop ‘feeding up’ a baby to get them to put on weight, and to move to thinking about ‘weight maintenance’ feeding instead. However, it was interesting to observe (again) that as each FGD progressed, parents started to express more uncertainty about their child’s weight status; represented by this quote:

*“I had a lot of pressure with Tom my eldest cause he was so very skinny, people were like ‘you must feed him’ .. And then my mum… well everybody just seemed to be going ‘he’s too skinny’, so we started on… we were at a party or something and he got some chicken nuggets or something like that and he liked those and he liked sausage rolls, and I started… and I found it wasn’t till he was 18 months old I realised I was giving him all these things to try and fatten him up, but actually I thought I’ve got to get away from that otherwise he’s never going to get out of the habit of eating like that.”* (P20)

These later discussions also pointed to parents’ uncertainty about the point that they thought they should start being concerned about their child being overweight (as opposed to worrying about them putting on enough weight) and, about the measures they should or would use once their child had got beyond the baby stage to make this judgement. Anxieties were also expressed about managing a slightly older infant’s food intake when they seemed excessively hungry, once they had outgrown the baby stage. Some expressed concerns about withholding food from their children when they were demanding food outside of meal times. The dilemma mothers seemed to face was their concern not to ‘give in’ to those demands, causing their child to put on too much weight, yet at the same time, fearing that their child might genuinely need the extra nourishment to help them deal with ‘growth spurts’ that were not always obvious at the time, illustrated by this quote:

*“I don’t really have any questions about my little girl. I do feel that both of my children have appetites, you know, but… my little boy I would say he does seem chunkier, but I think he goes through phases, you know, it’s almost like he seems a bit chunky but then he must have a growth spurt and then all of a sudden he’s sort of, you know, back to normal again really [laugh], but no they’re both solid my children, but I do home cook quite a lot, yeah, and my children like to be fed [laugh]! “(P25)*

#### Managing meals: managing bodies

The challenge of feeding siblings in the same family with different appetites, different food tastes and different body composition (and energy requirements) was also widely discussed. A commonly presented scenario was parent’s puzzlement at the similar intakes of their children but their different body sizes; e.g. with one child apparently staying slim while the other became overweight while eating the same amount of food, highlighted by this example:

*“I think my son might be different, that’s what I’m worried about my son, you know, when she went to P1, my daughter and son are so different, you know, my daughter she can eat all the fruit under the sun, aye my son (xxx name of son) he’s very select in what he’ll eat, you know, I do try and get him to eat his fruit a day but, you know, it’s hard work, and he’s such a different build to his sister, I mean, he’s solid he really is and anybody that picks him up is like… I don’t think he’s fat but he is solid and he’s always weighed a lot and I’m dreading him going for his P1 assessment if she came back obese, I dread to think what he’s going to come back as*” (P11)

Concerns to avoid stigmatising the overweight child within the family, or to develop ‘food issues’ for the ‘normal’ weight child, were commonplace for participants with more than one child in our study.

Some parents also described the pressure they experienced in dealing with the wide range of food and meal preferences at family mealtimes. They reported managing this situation in two ways. Some talked about not being able, or prepared to cook more than one meal at each mealtime to accommodate different food preferences, and having rules in their home about cooking only one meal at mealtimes while others spoke about trying to cater to meet all tastes by cooking or producing a range of meals each mealtime;

“..*I remember thinking one day she said ‘no I’m not eating that’ and thinking well she had that last week, absolutely fine, and I remember thinking to myself [clicking of fingers sound] ‘that’s fine not to eat it’ and I thought to myself ‘she’s not going to die, she’s not going to eat that meal, that’s fine, don’t eat it’ but what I wasn’t prepared to do is to start cooking three or four different meals and I’ve seen my friends do it, and I thought I can’t do this, … I’m not doing the separate meals for us all, she can eat…” (P19)*

#### Undermined by others

The food values and practice of other family members also represented a significant challenge to participants’ healthy feeding endeavours. Some described holding conflicting views compared to their partners or husbands (ex and co-habiting) about children’s portion sizes. Fathers (particularly those who were away from their children for long periods because of work or who were separated from the mother) were portrayed as prone to giving their children sweets and junk food treats. This was viewed as undermining the ‘hard work’ done in teaching and helping their children develop healthy eating habits by those who highlighted it.

*“Well me and my husband have split up now but no Graham he’s the same, he would eat… he’s a lorry drive, stop and get fry ups and, you know, I’d try and provide healthy food and he would just happily have fry ups and stuff like that. And same like, he would just cook that for the kid” (P30)*

One mother talked in detail about her ex-husband relying on convenience foods and fast food restaurants to feed their children when they were with him at weekends. She described her surprise and frustration in finding that her children were very familiar with the layout of their local MacDonald’s during one of her (rare) visits to the establishment with them.

Grandparents were also described in a problematic way. A few parents talked about their own parents giving their (grand) children the ‘wrong’ type of food as treats. A minority expressed frustration at their parents’ ‘double standards’ regarding bringing up children, as they perceived themselves as being given very limited access to these types of foods as children.

*“they get so much sweets when they go to grannies and I says to my husband, you know, ‘you weren’t brought up like that’ and, you know, they had a sleepover one night and they didn’t have I think it was coco pops, you know, so granddad went away into the shop and got coco pops for them before breakfast in the morning! You know, sorry, you don’t do that, you would never have brought your own children up like that” (P5)*

## Discussion

This study points to a number of key challenges for policy makers and professionals concerned with developing interventions or programmes intended to prevent infants becoming overweight or obese. Those challenges lie in being able to engage parents’ interest in prevention and weight management for under fives in the face of their strong sense of personal responsibility and ownership about this issue, and, their concomitant, general scepticism about health professionals’ roles and clinical measures of overweight and obesity in assisting them to manage their child’s weight. However, it also seems clear that mothers are attempting to get the ‘balance right’ for their child in the face of multiple intrinsic uncertainties associated with infant feeding, and, a wide range of external challenges to their child weight management intentions.

Our data indicates that mothers view the primary cause of childhood obesity as one of parental failure, and in particular, failure to manage a child’s food intake. This ‘individualised’ parental deficit explanation of obesity is consistent with research conducted elsewhere. For example, qualitative research conducted with UK adults (who self-identified as overweight or obese) revealed a dominant view of personal failures (behavioural and genetic) as the main causes of their problem. This contrasted with health professionals’ and policy makers’ views generated within the same study, that considered environmental, economic and social factors the more powerful explanation of the same [18].

Indeed, the volume of discussion that emerged about parent’s central role here, suggests that participant’s identities - as capable agents- are heavily invested in their child’s weight status. This view concurs with other research with mothers in Australia who believed they were judged, according to their child’s body size. All were keen to communicate their ‘weight management’ competence to us; a finding that was also consistent with Canadian research [19]. Yet existing evidence suggests that there is little association between parenting style and children’s dietary intake [20]. If anything, there is some suggestion that food restriction may have negative consequences [21, 22]. Furthermore, it is argued that countervailing social, economic and cultural factors interfere with parent’s decisions and ability to help their children manage their weight [1], something that was also evident in our data. Such factors are implicated in the more powerful explanation of adult and childhood obesity trends today [23–26]. Indeed, The UK Government Office of Science drew attention to the wide range of factors (and the multiple negative feedback loops linking them) associated or implicated in the rise of obesity levels globally, with the publication of their Full Systems Obesity Map in 2007 [27]. This report concluded that “*the overwhelming scientific consensus that modern life has become a major driver of obesity*…[and that] *.. that individual responsibility is important but insufficient to tackle obesity on its own”.*

The association between parental identity and child body weight has also been suggested as a partial explanation as to why parents strongly resist the idea of labelling under five year olds as overweight and obese [28, 29]. There was a strong perception in this study that not only would study participants not use a weight management programme targeting their children on the basis that it would call their own competence into question, but that other parents would also be put off from engaging with such a programme. Our participants were also deeply sceptical about the current clinical measures used to assess child weight status. This finding is consistent with previous research that has explored parental perspectives regarding this issue, which has also found parental views about child weight status to be at odds with clinical professional perspectives about the issue [30–32]. And according to orthodox clinical measures, parents are highly likely to misclassify their child’s weight status [31, 33–35]. However, it seems that health professionals are apparently as guilty as the general public in misclassifying children’s weight status or their own weight status [33, 36]. Furthermore, our participants reported receiving differing and conflicting advice from the primary health care professionals they had consulted or were referred to for weight management advice – which may or may not have been the case. Given the ongoing controversy amongst the scientific community about the relevance and legitimacy of the BMI scores [37, 38], it is perhaps no surprise that parents are sceptical of professional advice.

Whether or not parents in this study did or did not have a child with a weight problem, mothers in this study simply did accept it as a problem needing some form of resolution *at this stage*. A number of psychosocial models of health behaviour change include and emphasise the need for individuals to have perceived an issue as a problem in order for them to be willing to take action [39]. Therefore, it seems unlikely that parents would be willing to engage with a weight management programme on an issue they did not perceive to be, or were willing to acknowledge, as a problem at this point.

The mothers who took part in this study were concerned that their children should reach and maintain an optimal weight in the future, but believed that this could be achieved based on their endeavours that were focused on the apparently simple notion of ‘getting the balance right’. However, this ‘balancing’ appears to be anything but simple, and indeed, seems a highly complex act with the odds, we would argue, stacked in favour of weight gain, a view that is supported by evidence presented by Levitsky and Pacanowski who concluded eating behaviour was highly conditioned subconsciously, by a wide range of extrinsic variables or ‘food primes’ that encourage individuals to consume excess energy, and gain weight [40]. Indeed, it is paradoxical that despite mothers’ strong sense of personal responsibility for their child’s weight and, scepticism about health professional intervention to help with the issue, they were obviously dealing with varying but significant degrees of challenge in relation to theirs and their children’s food practices and weight management intentions. This study suggests that these challenges emerged in two distinct areas, i.e. [1] intrinsic doubts held about appropriate infant portion sizes, and, knowing when to stop ‘feeding up’ for weight gain, and [2] extrinsic challenges to their intentions to stick to their ‘balancing’ rules/intentions.

Portion size confusion was evident not only in terms of mothers’ views about their children’s portion sizes, but was also present in their own experiences of eating outside the home. Indeed, environmental cues are key determinants of food intake and overconsumption according to [41] as Brownell puts it,” …*cheeseburgers, french-fries,..potato chips and cheese curls, once unusual, are as much our background as trees, grass and clouds”* [42]. Recent UK research has found that there has been an obvious ‘up sizing’ of portions (and associated caloric content) of processed foods in the UK since 1993 [43]. Furthermore, this study also found that people were confused about appropriate portion sizes, which was also a feature of our findings. And as well as portion size confusion, the uncertainty mothers in our study expressed about when to stop demand feeding or feeding for weight gain in the absence of a definitive signal, was also found in a study of French mothers’ experiences of infant weaning [44].

It was also striking that children and families’ food behaviour was the dominant locus of mother’s accounts of child weight management, and that this had occurred despite our probing for their views on the role of physical activity in discussions where this did not naturally emerge. We found mothers’ tendency to focus on the energy side of the energy balance equation, particularly interesting given the emphasis placed (one might argue, by society) on physical activity as a key weight management strategy exemplified by the media (see for example [45], and, by governments (see for example the portfolio responsibilities for the Scottish Government Minister for the Commonwealth Games [46], and, the downplaying by the food industry of the role that high caloric foods play in weight gain [47–50]. It was also interesting to observe our participants tendency to focus on food consumption and behaviours rather than physical activity, given the emerging evidence and controversy surrounding the complex and (some would argue) limited role that physical activity plays in weight reduction [51–54].

On top of these intrinsic doubts, the ‘busyness” and complexity of our participants’ family lives starkly emerged during the focus groups, as mothers described their weight management efforts. It seems many were devoting substantial energy to meeting highly variegated family food preferences and requirements. During an era where the cultural norm (in the West at least) is to expect and demand choice, and to have all individual needs and desires met [55], it is perhaps not so surprising to have encountered these accounts of family meal management. Willmot & Nelson argue that the rise of the so-called ‘democratised family’ and ‘personalisation of consumption’ makes shopping for the family as a unit a thing of the past. They suggest this process is more about combining lists of individual wishes, making the practice of procuring family meals a more complicated and time-consuming endeavour.

It was also interesting to find, despite earlier portrayals of themselves as able ‘weight managers’, mothers talking honestly about cutting corners and breaking their own food rules when tired or pressed for time. These experiences were also evident amongst participants in Carrigan’s study of mothers in Birmingham. It is argued that employed UK parents with children experience greater time pressure than those without children [56, 57]. Carrigan et al. also draws attention to the dilemmas faced by mothers trying to manage meals in busy households with ‘unconventional work hours and meal schedules’. Indeed [58] study of low wage mothers in the USA found, while mothers valued and aspired to providing high nutritional quality food, this became less important when it became necessary to get (any) food on the table as quickly as possible, so they could complete other necessary caring or employment-related commitments [59]. Also found that parents in France and UK commonly reported lacking time to ‘cook from scratch’ despite a desire to do so, and resorted to a mix of raw and convenience type food as routine meal practice. Rapport cited in [60] maintained that current global working patterns have created less time for the care of children, older people and communities. It was estimated, for example, that American parents were spending an extra month a year working in 1987, compared to 1969 [61] (citing Schor 1991) (page 116) as consequence of their spending more hours at work in combination with the time spent on domestic tasks that had not declined in line with those increases to outside of home working time. These trends have led to a rise in the numbers of working women with children undertaking the so-called “double shift” to accommodate paid and domestic work. Over the same period, there has been a dramatic decline in the amount of time spent in UK homes on cooking and eating food (estimated by Hughes in [62] l to have fallen from an average of 2.5 hours in 1934 to an average of 10 minutes in 2010).

Skelton argues that prevailing approach to paediatric obesity treatment (both practice and research) fails to adequately factor in a comprehensive understanding of the family’s functioning and processes, and that this might explain why clinician’s advice to implement apparently simple, high impact behaviour change, routinely fails to be implemented by those receiving the advice [63]. Furthermore, as Jabs (2007) argues, nutritional advice typically focuses on what to eat, but seldom on how to fit those recommendations into busy lives [58].

It is also important to draw attention to the presence of apparently conflicting food values within families that emerged here, and the feeling that mothers were expressing about their weight management efforts being undermined by others. A recent review found that the literature on father’s child feeding practices is scant, but that, from what little evidence did exist, it seemed that there were differences in mothers and fathers’ feeding practices with fathers more likely to pressurise their children to eat and less likely to monitor children's food intake compared with mothers [64]. Furthermore, the review suggested that child adiposity and a range of child and parent characteristics were also associated with fathers’ feeding practices. And while mothers have been the primary focus of childhood nutrition research on the basis of historic perception of them as primary care givers [64], it is clear that huge changes have occurred in the way fathers are spending time and interacting with their children. Feinberg for example has drawn attention to the different models of parenting that have emerged, in response to women’s increased participation in the labour market since the 1980s [65]. It would be naive for those developing weight management interventions to ignore or downplay the role and influence of fathers in their endeavours.

As already highlighted, we failed to recruit parents on very low income. The economic constraints faced by those families are likely to produce a range of additional weight management challenges, related to food insecurity. For example, food prices have risen by 30.5% in the UK in the last five years, which is two and a half times the rate of increase in the National Minimum Wage [66]. During the same period, there has been a general decline in household food expenditure in the UK, with a marked shift towards the consumption of cheaper, more energy dense foods [67]. This is particularly marked in households with very young children and is an area which requires further investigation. In addition, we did not interview any fathers or grandparents as intended. On reflection, recruitment through nursery schools was probably not the best place to reach men or grandparents, and this area requires further investigation through other avenues, given the family tensions alluded to in this study. Furthermore, a relatively small number of parents took part in this study, and therefore the results are not simply generalisable. However, we believe that the results are conceptually generalisable [17] to parents in other parts of the UK.

## Conclusion

The results of this work suggest that significant challenges exist for policy makers and health professionals concerned with developing ‘family-centred’ childhood weight management interventions for very young children. The challenge lies in the fact that mothers believe themselves to be the primary (and capable) agents of obesity prevention in the early years – but who are, at the same time, attempting to deal with many mixed and conflicting messages and pressures emanating from their social and cultural environments that tend toward weight gain.

Participants in this study did not perceive themselves to be lacking information about healthy eating. Indeed all those who took part portrayed themselves as very well informed about this issue. Furthermore, our study participants’ views of themselves as capable parents, was very closely associated their child’s weight status. We think it likely that many parents would avoid engaging with such programmes, particularly if they did not consider their child had an obvious weight problem. However, parents may be more willing to engage with a programme aimed at helping parents cope with the struggles and challenges that they face day-to-day in raising a healthy weight child in the context of complex family life. For example, that acknowledges the uncertainties and difficulties that exists in recognising when to stop ‘demand feeding’ their infants - the ‘gold standard’ advice for neonatal and early infant feeding in the UK - and to switch to providing sufficient amounts of food that does not cause excessive weight gain. Or, that acknowledges that families are not homogenous units (as they are often characterised in health promotion programmes) and that different food values and practices will exist within them that can make it difficult for individual parents to follow through on good ‘weight management’ intentions or directions from health professionals. Crucially, programmes must take account of the sensitivities and vested interests that exist amongst parents about this issue.

And despite the antagonism that parents expressed about a weight management intervention for infants, the time could be right for professionals and policy makers to be more radical and innovative in developing (policy/programme) interventions in collaboration with parents (as opposed to dealing with them as consultees in the delivery of health care services), given that both mothers and health professionals are concerned with achieving the same goal, either now or for the future. Perhaps a starting point for this work would be the creation of more honest policy and public discourse about the sheer complexity and challenges involved with achieving healthy weight gain in early childhood in an era where excessive consumption of cheap (ubiquitously available) energy dense foods is the norm. Perhaps engaging in such a debate with parents may also help to raise awareness and gain popular support for (non-health care) policy interventions that many argue are required to address the structural causes of obesity - that would have greater impact at a population level, and which are less likely to generate or magnify current obesity-related health inequalities [32, 68–70].
